# The nestin-expressing and non-expressing neurons in rat basal forebrain display different electrophysiological properties and project to hippocampus

**DOI:** 10.1186/1471-2202-12-129

**Published:** 2011-12-20

**Authors:** Jianhua Zhu, Huaiyu Gu, Zhibin Yao, Juntao Zou, Kaihua Guo, Dongpei Li, Tianming Gao

**Affiliations:** 1 Department of Anatomy and Neurobiology, Zhongshan School of Medicine, Sun Yat-sen University, Guangzhou, China; 2 Department of Neurobiology, Southern Medical University, Guangzhou, China

## Abstract

**Background:**

Nestin-immunoreactive (nestin-ir) neurons have been identified in the medial septal/diagonal band complex (MS/DBB) of adult rat and human, but the significance of nestin expression in functional neurons is not clear. This study investigated electrophysiological properties and neurochemical phenotypes of nestin-expressing (nestin+) neurons using whole-cell recording combined with single-cell RT-PCR to explore the significance of nestin expression in functional MS/DBB neurons. The retrograde labelling and immunofluorescence were used to investigate the nestin+ neuron related circuit in the septo-hippocampal pathway.

**Results:**

The results of single-cell RT-PCR showed that 87.5% (35/40) of nestin+ cells expressed choline acetyltransferase mRNA (ChAT+), only 44.3% (35/79) of ChAT+ cells expressed nestin mRNA. Furthermore, none of the nestin+ cells expressed glutamic acid decarboxylases 67 (GAD_67_) or vesicular glutamate transporters (VGLUT) mRNA. All of the recorded nestin+ cells were excitable and demonstrated slow-firing properties, which were distinctive from those of GAD_67 _or VGLUT mRNA-positive neurons. These results show that the MS/DBB cholinergic neurons could be divided into nestin-expressing cholinergic neurons (NEChs) and nestin non-expressing cholinergic neurons (NNChs). Interestingly, NEChs had higher excitability and received stronger spontaneous excitatory synaptic inputs than NNChs. Retrograde labelling combined with choline acetyltransferase and nestin immunofluorescence showed that both of the NEChs and NNChs projected to hippocampus.

**Conclusions:**

These results suggest that there are two parallel cholinergic septo-hippocampal pathways that may have different functions. The significance of nestin expressing in functional neurons has been discussed.

## Background

Medial septal/diagonal band complex (MS/DBB) is a highly heterogeneous region with different types of neurons and implicated in various functions such as arousal, sensory processing, motivation, emotion, learning and memory [1-3]. MS/DBB contains cholinergic, GABAergic neurons, glutamatergic neurons [4-7], nitric oxide synthase positive neurons, and a number of peptidergic neurons [8] that co-localize with GABAergic or cholinergic neurons [9]. Cholinergic neurons have received particular attention not only for their roles in learning and memory, but also for their involvement in the pathology of Alzheimer's disease (AD) [10,11].

There are four classes of neurons in the MS/DBB distinguished by electrophysiological characteristics [4,12-15]. The first group includes slow-firing neurons with broad action potential (AP) and long duration afterhyperpolarization (AHP). The second group consists of fast-firing neurons with narrower action potential and shorter AHP. The third group comprises burst-firing neurons whose membrane properties are similar to those of fast-firing neurons, but can fire in bursts when depolarized from a hyperpolarized holding potential (-75 mV or -80 mV). A recent study confirmed that the slow-firing neurons are cholinergic, and both of the fast-firing and the burst-firing neurons are GABAergic neurons. The fourth class of neurons is cluster-firing neurons and is glutamatergic. These neurons have electrophysiological properties similar to those of slow-firing neurons. However, prolonged (4s) depolarization revealed that these neurons exhibited a cluster-firing pattern [4]. Huh, et al. [16] revealed that the glutamatergic neurons in MS/DBB display a highly heterogeneous set of firing patterns including fast-, cluster-, burst-, and slow-firing, therefore, electrophysiologic properties of the glutamatergic neurons in MS/DBB should be further studied.

Nestin is an intermediate filament protein expressed transiently by neural progenitor cells and reactivated glial cells [17] and is involved in cell survival and reparation [18]. Recently, researchers identified a group of nestin immunoreactive (nestin-ir) cells in the MS/DBB of adult rats and humans [8,19,20]. The expression of neuron specific enolase (NSE) and neuron-specific nuclear protein (NeuN), but not glial fibrillary acidic protein (GFAP), suggests that the nestin-ir cells are functional neurons. They are also similar to cholinergic neurons in distribution and morphology and are intermingled with other types of neurons. Double labelling immunohistochemistry showed that there was no overlap between nestin-ir and parvalbumin immunoreactive (PV-ir) neurons in the MS/DBB, and about 35% of nestin-ir neurons were choline acetyltransferase immunoreactive (ChAT-ir) neurons [8]. Further study showed that progressive degeneration of nestin-ir neurons might be involved in the mechanisms of aging and memory deficit [21]. Although a few basic morphological studies have been made on nestin-ir neurons, the neurochemical properties of nestin-ir neurons and the significance of nestin expression in functional neurons remain unclear. The purpose of the present study is to explore the neurochemical properties of nestin-expressing (nestin+) neurons with single-cell RT-CPR (sc-RT-PCR), to investigate the intrinsic membrane properties and excitatory synaptic afferent currents of nestin+ neurons using whole-cell patch clamp recording, and to explore the neuronal circuit of nestin+ neurons with retrograde labelling combined with nestin and ChAT immunohistochemistry.

## Results

### Chemical phenotypes of MS/DBB neurons identified by sc-RT-PCR

A total of 106 Medial Septal/Diagonal Band Complex (MS/DBB) neurons were electrophysiologically recorded and their chemical phenotypes were identified by multiplex sc-RT-PCR. The results showed that the mRNAs encoding nestin, ChAT, glutamic acid decarboxylases 67 (GAD_67_), vesicular glutamate transporters 1 or 2 (VGLUT_1 _or VGLUT_2_) could be reversely transcribed and amplified from the harvested cytoplasm. Automatic sequencing confirmed that each PCR product is from the target cDNA. The MS/DBB neurons studied in our experiment were comprised of 79 ChAT mRNA-positive neurons (ChAT+) that are cholinergic neurons, 13 GAD_67 _mRNA-positive neurons (GAD_67_+) that are GABAergic neurons. There were 11 neurons co-expressing ChAT mRNA and GAD_67 _mRNA, of which, 6 were categorized as cholinergic neuron and 5 as GABAergic neuron according to their electrophysiological properties. Nine neurons solely expressed VGLUT_1 _mRNA or/and VGLUT_2 _mRNAs but not ChAT mRNA or GAD_67 _mRNAs, which confirms the identification of glutamatergic neurons [4,22]. Among the 40 nestin mRNA-positive (nestin+) neurons, 87.5% (35/40) neurons expressed ChAT mRNA. Conversely, 44.3% (35/79) of ChAT+ neurons expressed nestin mRNA. However, no nestin mRNA was found co-expressing GAD_67 _mRNA or VGLUT mRNA. The neurons did not express any of these mRNA were discarded.

### The intrinsic membrane properties of nestin mRNA+ neurons

**Figure 1:**
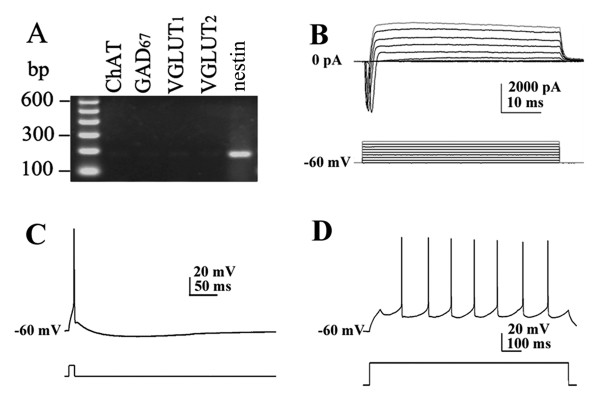
**Nestin mRNA-positive cells in MS/DBB are functional neurons**. A. Agarose gel analysis of the sc-RT-PCR products obtained from a single MS/DBB cell. The only PCR-generated fragment was nestin. B. Whole cell current of the same cell depolarized from -60 mV to 10 mV in voltage-clamp mode showed characteristic of functional neurons. C. The cell shows typical neural action potential when depolarized to the threshold potential. D. A sustained depolarizing current (1000 ms) elicits a train of repetitive action potentials. The membrane responses were elicited using positive currents of 0.2 nA from -60 mV in panel C and D.

**Figure 2:**
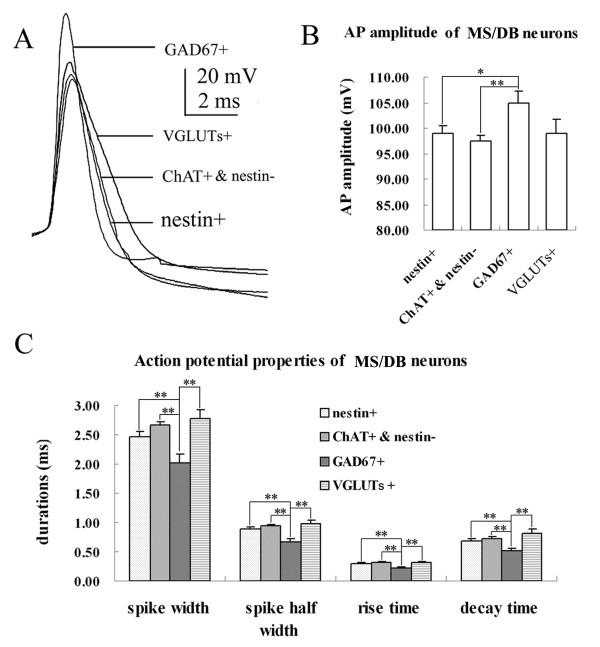
**Comparison of action potential properties of MS/DBB neurons**. A. Representative action potentials from all cell types (superimposed) show the differences in spike shape and width among the four cell types. GAD_67_+ neurons have the narrowest action potentials, where as VGLUT+ neurons have the broadest action potentials. B. Histogram of action potential amplitude of all cell types. C. Histogram of spike parameters of all cell types. ^* ^*P *< 0.05;** *P *< 0.01.

**Figure 3:**
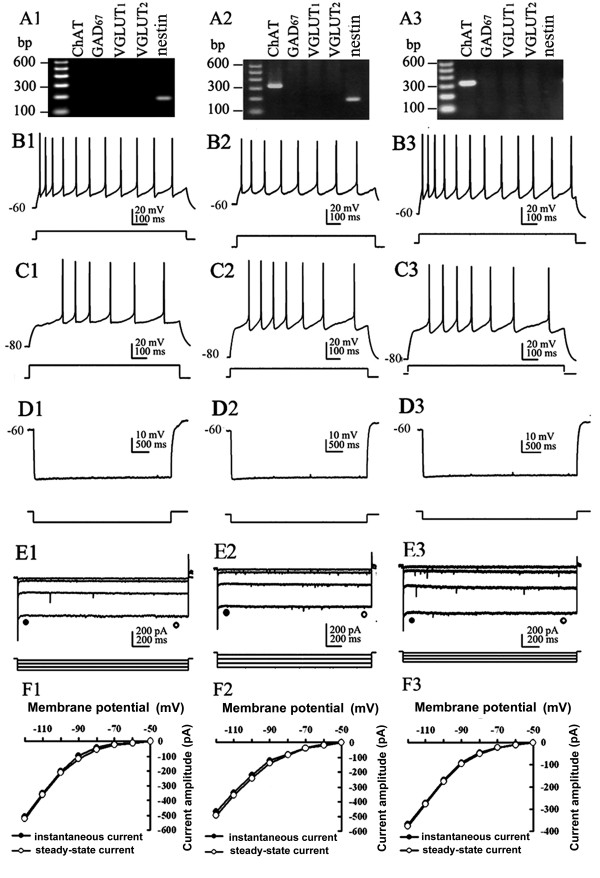
**Electrophysiological properties of nestin+ and/or ChAT+ neurons**. A1-F1: Nestin+ & ChAT- neurons, A2-F2: Nestin+ & ChAT+ neurons, A3-F3: Nestin- & ChAT+ neurons. A1-3: Agarose gel analysis of the sc-RT-PCR products to identify chemical phenotypes of recorded neurons. B_1-3 _and C1-3: In current-clamp mode, membrane responses of the same MS/DBB neuron to depolarizing current pulses (0.2 nA) applied from membrane potential of -60 mV (B1-3) or -80 mV (C1-3). All neurons display slow-firing activity. D1-3: Injection of a hyperpolarizing current pulse from -60 mV, no depolarizing sag and rebound firing were found in each kind of neuron. E1-3: Hyperpolarizing voltage steps applied from a holding potential of -50 mV showed absence of conspicuous *I*_h _in all groups of neurons. F1-3: I-V plots of instantaneous (filled circle) and steady-state (open circle) current derived from the data in E1-3. The *I*_h _of nestin+ & ChAT+ neuron was mildly larger than that of nestin- & ChAT+ neuron (E2, E3, F2, F3).

**Figure 4:**
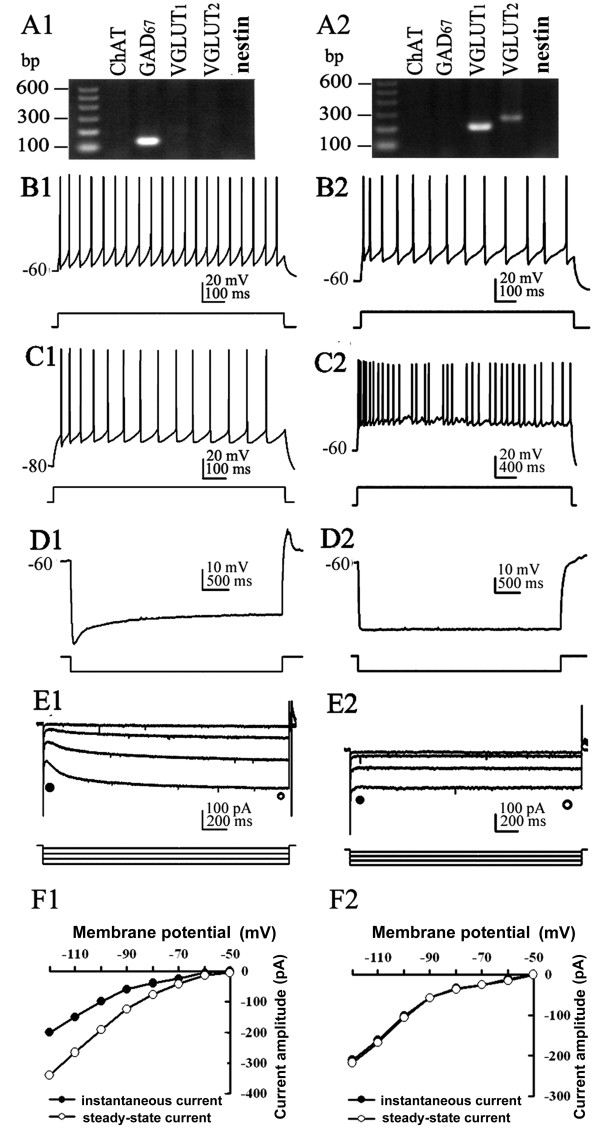
**Electrophysiological properties of GAD67+ and VGLUT+ neurons**. A1-F1: GAD_67_+ neuron presents electrophysiological properties of fast-firing neuron. A2-F2: VGLUT+ neuron presents electrophysiological properties of cluster-firing neuron. A1-2: Agarose gel analysis of the sc-RT-PCR products to identify chemical phenotypes of recorded neurons. B1 and C1: Membrane responses of the same MS/DBB neuron to injection of depolarizing current pulses applied from a membrane potential of -60 mV (B1) or -80 mV (C1). GAD_67_+ neuron displays fast-firing activity. B2 and C2: Membrane responses of the VGLUT+ neuron to injection of depolarizing current pulses (0.2 nA) applied from a membrane potential of -60 mV, prolonged depolarization current (4s in duration) elicits cluster-firing separated by subthreshold membrane oscillations (C2). D1-2: In current-clamp mode, the response to injections of hyperpolarizing current pulse from -60 mV, note profound depolarizing sag in GAD_67_+ neuron, but no rebound firing action potential (D1). E1-2: Currents recorded in voltage-clamp mode evoked by a series of hyperpolarizing voltage steps applied from a holding potential of -50 mV. Notice the profound depolarizing amplitude inward current in GAD_67_+ neuron, as shown by the differences between the amplitudes of the instantaneous current (filled circle) and steady-state current (open circle). F1-2: Instantaneous and steady-state I-V plots derived from the data in E1-2.

**Figure 5:**
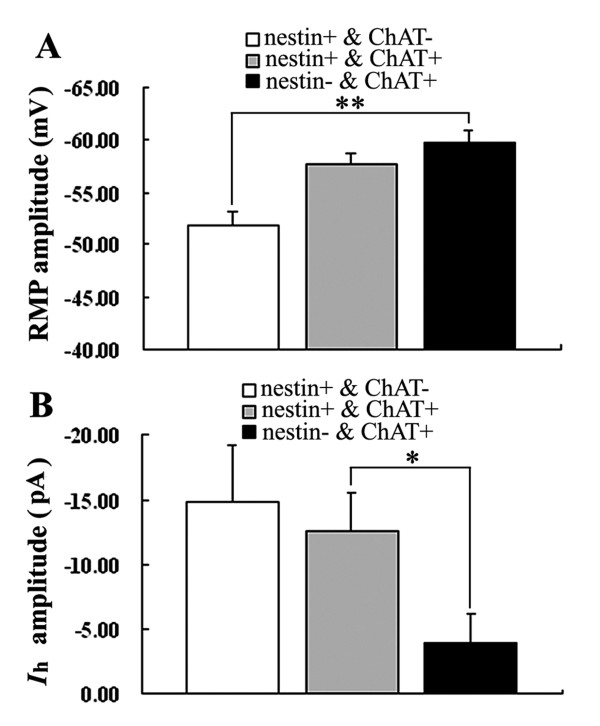
**Comparison of RMP and *I*_h _amplitude of nestin+ and/or ChAT+ neurons in MS/DBB**. * *P *< 0.05; ** *P *< 0.01.

Eighty-seven neurons were assessed for their electrophysiological profile. This assessment identified 69 slow-firing neurons, 5 cluster-firing neurons and 13 fast-firing neurons. Although some neurons presented rebound action potentials following a hyperpolarization current injection, we did not find any typical burst-firing neuron in our experiment. All of the recorded nestin+ cells were excitable, with typical electrophysiological characteristics of functional neurons. In voltage-clamp mode, when depolarized from -60 mV, typical neuronal whole-cell currents (comprised of large inward Na+ current and outward K+ current) could be elicited (Figure 1B). In current-clamp mode, typical neural action potential was observed in response to a short period of depolarization, and repetitive action potentials could be elicited when sustained positive current was applied (Figure 1C, D). As most of nestin mRNA co-expressed with ChAT mRNA, we first compared electrophysiological properties of nestin+ neurons (including nestin mRNA-positive & ChAT mRNA-negative neurons and nestin mRNA-positive & ChAT mRNA-positive neurons) to nestin mRNA-negative & ChAT mRNA-positive (nestin- & ChAT+) neurons, and with GAD_67_+ neurons and VGLUT+ neurons (Table 1, Figures 2, 3 and 4). Then, we compared electrophysiological properties of nestin mRNA-positive & ChAT mRNA-negative (nestin+ & ChAT-) neurons, nestin mRNA-positive and ChAT mRNA-positive (nestin+ & ChAT+) neurons, and nestin mRNA-negative & ChAT mRNA-positive neurons (nestin- & ChAT+) so as to identify the electrophysiological characteristics among different categories of nestin and ChAT expressing patterns (Figures 3 and 5).

**Table 1:** Electrophysiological properties of chemically identified neurons in the rat medial septal/diagonal band complex

	nestin+	nestin- & ChAT+	**GAD**_**67**_**+**	VGLUT+
number of neurons	33	32	13	9
Cm (pf)	55.76 ± 3.05 ^b^	49.60 ± 2.37 ^b^	76.99 ± 9.60	43.58 ± 3.92 ^b^
Rm (MΩ)	400.56 ± 26.86	387.26 ± 27.27	289.63 ± 44.25	570.07 ± 120.25
Tau (ms)	0.85 ± 0.07	0.70 ± 0.05^a^	1.27 ± 0.18	0.64 ± 0.06 ^a^
RMP (mV)	-56.73 ± 1.05	-59.75 ± 1.14	-55.62 ± 1.91	-58.00 ± 3.47
AHP duration (ms)	223.77 ± 12.08 ^b^	230.52 ± 8.48 ^b^	165.23 ± 7.04	238.17 ± 16.97 ^a^
AHP amplitude (mV)	4.49 ± 0.21 ^b^	5.02 ± 0.35 ^b^	3.27 ± 0.25	4.80 ± 0.28 ^b^
AP amplitude (mV)	99.04 ± 1.37 ^a^	97.39 ± 1.22 ^b^	104.97 ± 2.22	98.85 ± 2.82
Spike half width (ms)	0.88 ± 0.04 ^b^	0.94 ± 0.03 ^b^	0.67 ± 0.06	0.99 ± 0.05 ^b^
Spike width (ms)	2.46 ± 0.09 ^b^	2.66 ± 0.06 ^b^	2.02 ± 0.14	2.78 ± 0.14 ^b^
rise time (ms)	0.30 ± 0.01 ^b^	0.32 ± 0.01 ^b^	0.23 ± 0.02	0.32 ± 0.01 ^b^
rise slope (mV/ms)	121.91 ± 8.30	105.85 ± 4.99 ^a^	196.45 ± 24.41	106.31 ± 8.06 ^a^
decay time (ms)	0.69 ± 0.03 ^b^	0.73 ± 0.03^b^	0.51 ± 0.05	0.82 ± 0.06 ^b^
decay slope (mV/ms)	-49.57 ± 3.21 ^a^	-43.69 ± 1.81 ^b^	-83.06 ± 9.41	-38.98 ± 2.55 ^b^
MF (Hz)	8.39 ± 0.45 ^b^	8.19 ± 0.49 ^b^	18.38 ± 1.73	9.22 ± 0.97 ^b^
F_MAX _(Hz)	20.73 ± 2.07 ^a^	18.86 ± 1.77 ^b^	28.99 ± 1.93	19.83 ± 0.82 ^b^
F_STEADY _(Hz)	6.86 ± 0.53 ^b^	6.67 ± 0.35 ^b^	17.23 ± 1.66	7.01 ± 0.49 ^b^
spike adaptation	0.55 ± 0.05 ^b^	0.56 ± 0.04 ^b^	0.28 ± 0.06	0.56 ± 0.11 ^a^
depolarizing sag (mV)	2.74 ± 0.62 ^b^	1.81 ± 0.32 ^b^	16.81 ± 0.77	2.22 ± 0.56 ^b^
*I*_h _(pA)	-12.90 ± 2.57 ^b^	-4.00 ± 2.17 ^b^	-186.82 ± 15.73	-3.49 ± 1.86 ^b, c^

Nestin+, nestin mRNA-positive neuron (including nestin mRNA-positive & ChAT mRNA-negative neurons and nestin mRNA-positive & ChAT mRNA-positive neurons); Nestin- & ChAT+, nestin mRNA-negative and choline acetyl transferase mRNA-positive neuron; GAD_67_+, glutamic acid decarboxylases 67 mRNA-positive neuron; VGLUT+, vesicular glutamate transporter 1 or/and 2 mRNA-positive neuron. Cm, membrane capacitance; Rm, membrane resistance; Tau, time constant; RMP, resting membrane potential; threshold, action potential threshold; AHP, afterhyperpolarization; MF, Mean firing rate; F_MAX_, maximal firing frequency; F_STEADY_, steady firing frequency. *I*_h_, hyperpolarization activated current of neurons when hyperpolarized from -50 to -120 mV. The current pulses used for membrane property analyses were 0.1-0.3 nA. All values are presented as means ± S.M.E. Significant differences are identified: ^a ^*versus *GAD_67_+ neurons, *P *< 0.05; ^b ^*versus *GAD_67_+ neurons, *P *< 0.01; ^c ^*versus *nestin+ neurons, *P *< 0.05.

Nestin+ neurons (including those neurons co-expressing ChAT mRNA) had a mean fire rate (MF) of 8.39 ± 0.45 Hz, maximal firing frequency (F_MAX_) of 20.73 ± 2.07 Hz, and steady firing frequency (F_STEADY_) of 6.86 ± 0.53 Hz. These neurons also had broad action potentials (spike width 2.46 ± 0.09 ms) and large afterhyperpolarization (duration 223.77 ± 12.08 ms, amplitude 4.49 ± 0.21 mV), which were similar to nestin- & ChAT+ neurons (*P *> 0.05). These data suggest that nestin+ neurons share many basic characteristics with nestin- & ChAT+ neurons in the MS/DBB. The spike width, AHP amplitude and duration of nestin+ neurons were significantly larger than that of GAD_67_+ neurons (*P *< 0.01). However, MF, depolarizing sag, and hyperpolarization activated current of neurons (*I*_h_) were smaller than those of GAD_67_+ neurons (*P *< 0.01). Furthermore, other key properties of nestin+ neurons were significantly different from those of GAD_67_+ neurons (*P *< 0.05, Table 1, Figure 2). Thus, the nestin+ neurons were distinctive from the classic GABAergic neurons. Nestin+ neurons shared some membrane properties of VGLUT+ neurons, but had larger *I*_h _and no cluster firing in response to prolonged depolarization from -60 mV. In summary, the *I*_h _of nestin+ neurons were smaller than those of the GAD_67_+ neurons, but greater than the *I*_h _of the VGLUT+ (Figures 3 and 4). Statistical analysis showed the different *I*_h _and other parameters among the subpopulations of neurons in MS/DBB (Table1).

Interestingly, while further analyzed the electrophysiological properties of nestin+ & ChAT-, nestin+ & ChAT+ and nestin+ & ChAT- neurons, we found that the *I*_h _of nestin+ & ChAT+ neurons were larger than those of nestin- & ChAT+ neurons (*P *< 0.05), and nestin+ & ChAT- neurons had a RMP of -51.80 ± 1.32 mV, which were significantly lower than that of nestin- & ChAT+ neurons (*P *< 0.01) (Figures 3 and 5). However, other electrophysiological differences (e.g., latency for first spike, slow after-hyperpolarizing potential, maximal frequency and action potential decay slope) among these neurons were not found.

### Excitatory postsynaptic currents recorded from nestin+ & ChAT+ neurons and nestin- & ChAT+ neurons

In this section, nineteen neurons, which contained 12 nestin- & ChAT+ neurons and 7 nestin+ & ChAT+ neurons, were recorded, and 3993 sEPSCs events and 3570 mEPSCs events were analyzed in total. The addition of 10 μM 6-cyano-7-nitroquinoxaline-2, 3-dione (CNQX, a non-NMDA glutamate receptor antagonist) abolished all synaptic events, indicating the involvement of non-NMDA glutamate receptors (data not shown).

**Figure 6:**
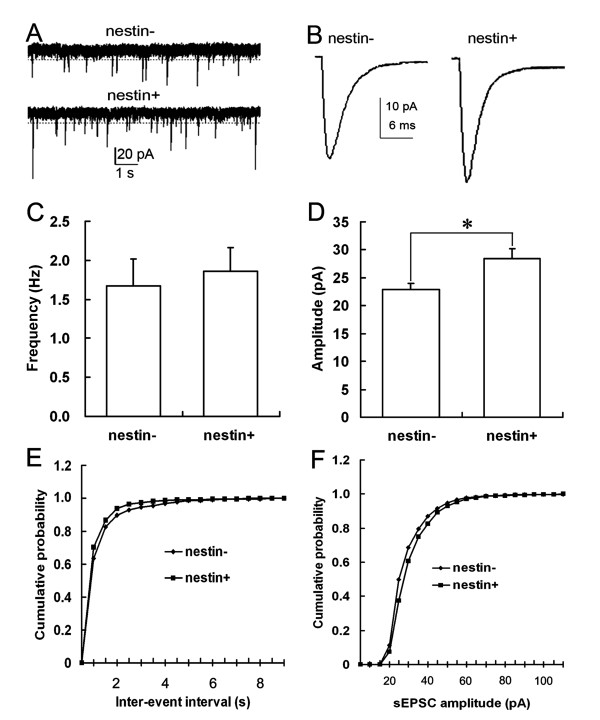
**Comparison of sEPSCs of MS/DBB nestin- and nestin+ cholinergic neurons**. A. Consecutive traces of sEPSCs recorded from MS/DBB nestin- and nestin+ cholinergic neurons. B. Average sEPSCs of nestin- and nestin+ cholinergic neurons. C. Comparison of the sEPSCs frequencies of MS/DBB nestin- and nestin+ cholinergic neurons. D. Comparison of the sEPSCs amplitudes of MS/DBB nestin- and nestin+ cholinergic neurons (*: *P *< 0.05). E. Cumulative probability distribution of sEPSCs inter-event intervals of nestin- and nestin+ cholinergic neurons in MS/DBB: the curve of nestin+ cholinergic neurons was on the left of the curve for nestin- cholinergic neurons (K-S Z = 2.644, *P *< 0.01); F. Cumulative probability distribution of sEPSCs amplitudes of nestin- and nestin+ cholinergic neurons in MS/DBB: the curve of nestin+ cholinergic neurons was on the right of the curve for nestin- cholinergic neurons (K-S Z = 4.549, *P *< 0.01).

The sEPSCs amplitude (28.45 ± 1.78 pA) of nestin+ & ChAT+ neurons was significantly larger than that of the nestin- & ChAT+ neurons (22.91 ± 1.05 pA) (student's *t-*test, two tails, *P *< 0.05). The sEPSCs amplitudes cumulative probability distribution curve of nestin+ & ChAT+ neurons showed a right shift compared to the curve for nestin- & ChAT+ neurons (K-S Z = 4.549, *P *< 0.01). This result suggests that the sEPSCs distribution patterns of nestin+ & ChAT+ neurons were different from those of nestin- & ChAT+ neurons. It also provided further evidence to confirm that the sEPSCs amplitude of nestin+ & ChAT+ neurons was significantly larger than those of the nestin- & ChAT+ neurons. Both the student's *t*-test and the Kolmogorov-Smirnov test (KS-test) were used to determine if the two datasets differ significantly. As student's *t*-test is a parametric test and may be more sensitive if the data meets the requirements of the student's *t*-test. The KS-test, on the other hand, has the advantage of making no assumptions about the distribution of data (non-parametric). Therefore, in order to compare the mean value and distribution of the sEPSCs and mEPSCs, we used both, finding that the KS-test is more suited than the student's *t*-test. The sEPSCs inter-event intervals cumulative probability distribution curve of nestin+ & ChAT+ neurons was on the left of the curve for nestin- & ChAT+ neurons (K-S Z = 2.644, *P *< 0.01), which indicates the sEPSCs frequency of the nestin+ & ChAT+ neurons was higher than that of the nestin- & ChAT+ neurons (Figure 6) [23,24].

**Figure 7:**
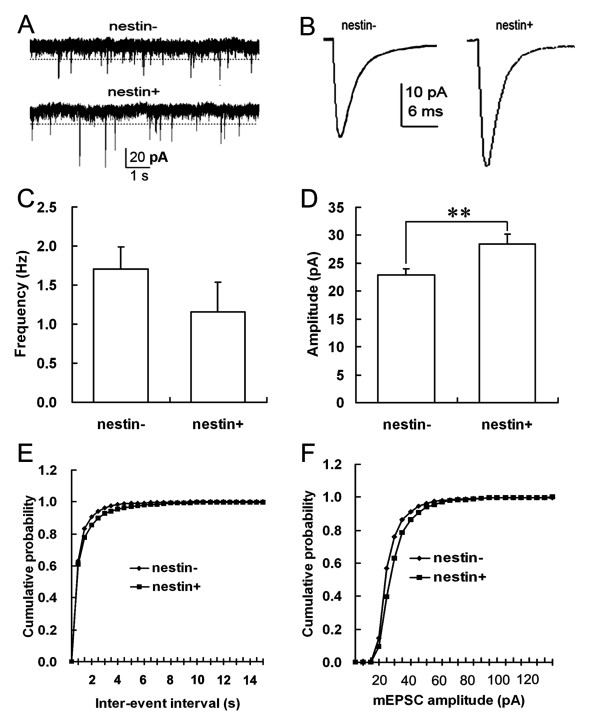
**Comparison of mEPSCs of MS/DBB nestin- and nestin+ cholinergic neurons**. A. Consecutive traces recorded from MS/DBB nestin- and nestin+ cholinergic neurons. B. Average mEPSCs of nestin- and nestin+ cholinergic neurons. C. Comparison of the mEPSCs frequencies of MS/DBB nestin- and nestin+ cholinergic neurons. D. Comparison of the mEPSCs amplitudes of MS/DBB nestin- and nestin+ cholinergic neurons (**: *P *< 0.01). E. Cumulative probability distribution of mEPSCs inter-event intervals of nestin- and nestin+ cholinergic neurons in MS/DBB: the curve of nestin+ cholinergic neurons was on the right of that of nestin- cholinergic neurons (K-S Z = 1.717, *P *< 0.01). F. Cumulative probability distribution of mEPSCs amplitudes of nestin- and nestin+ cholinergic neurons in MS/DBB: the curve of nestin+ cholinergic neurons was on the right of that of the nestin- cholinergic neurons (K-S Z = 8.217, *P *< 0.01).

In order to further explore the mechanism of the different sEPSCs between the nestin- & ChAT+ and nestin+ & ChAT+ neurons, mEPSCs of both kinds of neurons were studied. The independent samples student's *t*-test showed that the mEPSCs amplitude (29.01 ± 1.83 pA) of nestin+ & ChAT+ neurons was significantly larger than that of nestin- & ChAT+ neurons (22.64 ± 1.06 pA) (*P *< 0.01). The mEPSCs amplitudes cumulative probability distribution curve of nestin+ & ChAT+ neurons was on the right of that for the nestin- & ChAT+ neurons (K-S Z = 8.2165, *P *< 0.01). The mEPSCs inter-event intervals cumulative probability distribution curve of nestin+ & ChAT+ neurons was on the right for that of nestin- & ChAT+ neurons (K-S Z = 1.717, *P *< 0.01). These results confirmed that nestin+ & ChAT+ neurons had higher mEPSCs amplitude than nestin- & ChAT+ neurons and that the mEPSCs frequency of nestin+ & ChAT+ neurons was lower than that of the nestin- & ChAT+ neurons (Figure 7).

**Figure 8:**
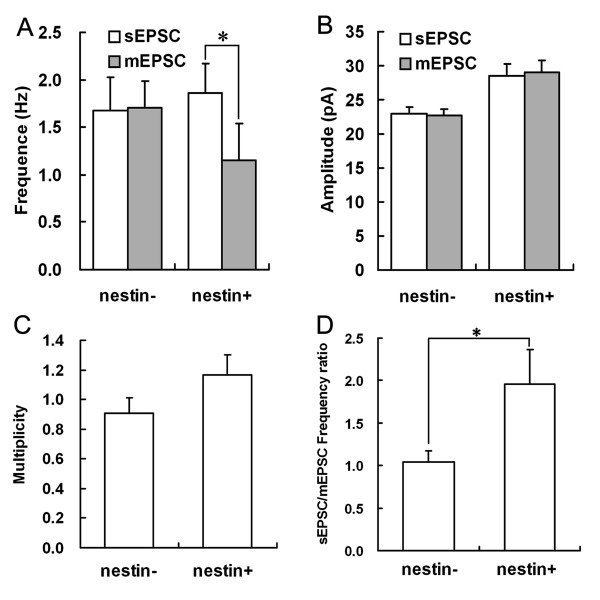
**Comparison of sEPSCs and mEPSCs of nestin- and nestin+ cholinergic neurons in MS/DBB**. A. Comparison of the frequencies of sEPSCs and mEPSCs of nestin- and nestin+ cholinergic neurons. B. Comparison of the amplitude of sEPSCs and mEPSCs of nestin- and nestin+ cholinergic neurons. C. Multiplicity of nestin- and nestin+ cholinergic neurons. D. sEPSCs/mEPSCs frequency ratio of nestin- and nestin+ cholinergic neurons. *: *P *< 0.05.

The paired samples student's *t*-test results showed that the mEPSCs frequency was significantly lower than sEPSCs frequency in nestin+ & ChAT+ (*P *< 0.05), but no difference was found between the mEPSCs and sEPSCs frequencies of nestin- & ChAT+ neurons (*P *> 0.05). The sEPSCs/mEPSCs frequency ratio of nestin+ & ChAT+ neurons was approximately two times higher than that nestin- & ChAT+ neurons. However, there was no difference between the amplitudes of mEPSCs and sEPSCs of nestin- & ChAT+ neurons or nestin+ & ChAT+ neurons (*P *> 0.05). These results suggest that the higher sEPSCs amplitude of nestin+ & ChAT+ neurons compared to the nestin- & ChAT+ neurons was not changed by 1 μM TTX, implied it might come from the higher excitability of nestin+ & ChAT+ neurons themselves rather than from stronger excitatory action potentials of presynaptic neurons. Furthermore, there was no difference between synaptic multiplicities of nestin+ & ChAT+ neurons and nestin- & ChAT+ neurons, which suggests that the nestin+ & ChAT+ neurons and nestin- & ChAT+ neurons shared similar maturity (Figure 8). In summary, these results provide powerful evidence that despite shared similar maturity, nestin+ & ChAT+ neurons receive stronger excitatory synaptic inputs and have higher excitability compared to nestin- & ChAT+ neurons.

### Immunofluorescence study of the biocytin-filled neurons

**Figure 9:**
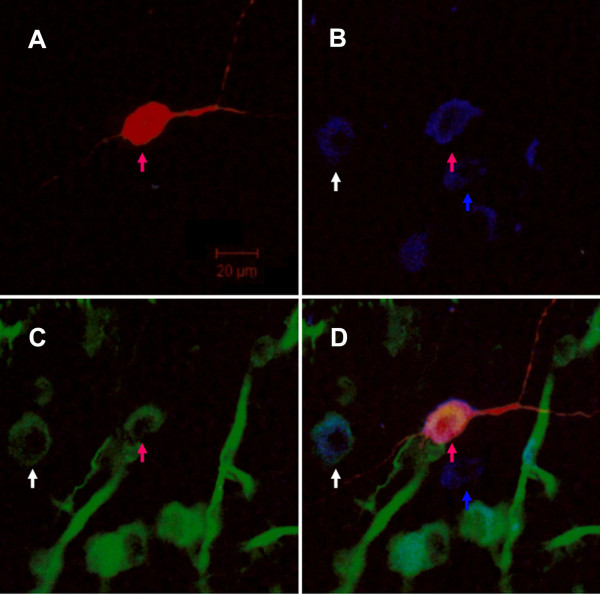
**Triple immunofluorescent study of biocytin-filled neuron**. A. Biocytin-filled neuron was visualized by rhodamine red-X-conjugated streptavidin. B and C showed ChAT- and nestin-immunoreactive neurons visualized by cy2 (blue) and alexa 405 (green) respectively. D. Image merged from A, B and C. The red arrow pointed to the cell double labelled by the nestin and ChAT antibodies and filled with biocytin by whole-cell patch clamp recording. The white arrow pointed to the cell double labelled by ChAT and nestin antibodies. The blue arrow pointed to a neuron that only expressed ChAT. Because the brain slice was made from neonatal rat and did not perfuse transcardially before slice preparation, there were some epithelium lining blood vessels labelled by nestin monoclone antibody in C and D. Scale bar was 20 μm.

Twenty-eight neurons were successfully filled with biocytin and visualized by Rhodamine Red-X. Cell bodies were particularly well-labelled, allowing us to determine their position relative to the MS/DBB. Biocytin-filled neurons were bipolar or multipolar and gave off two or three primary dendrites that subsequently bifurcated to the adjacent areas. The axons originated from the soma or proximal end of a primary dendrite. No evidence of axon collaterals was found in our slices. Of the 28 biocytin-filled neurons, 22 were ChAT-immunoreactive (ChAT-ir) neurons, among which 45.45% (10/22) were nestin-immunoreactive (nestin-ir) neurons. Eleven out of the 28 biocytin-filled neurons were nestin-ir neurons, 90.91% (10/11) of which were also ChAT-immunoreactive (Figure 9).

### Retrograde tracing of fast blue from the CA1 area of hippocampus

**Figure 10:**
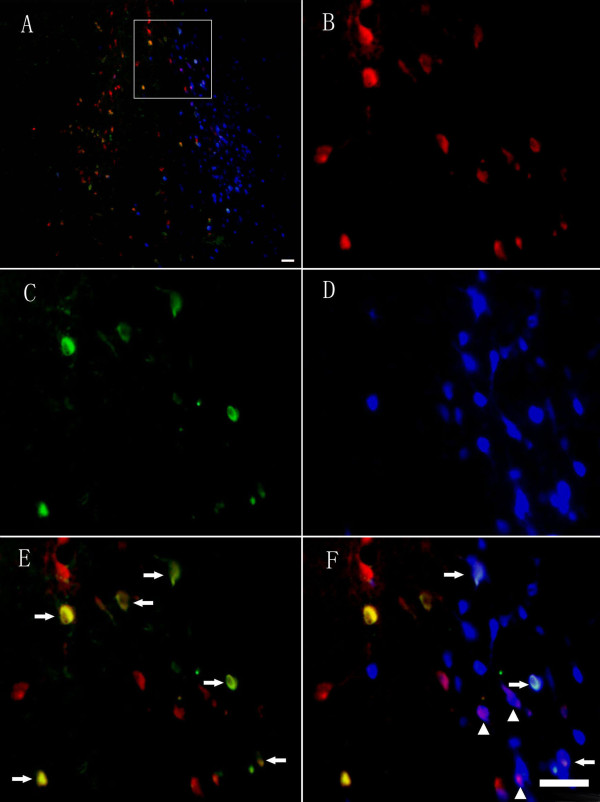
**Retrograde labelling demonstrates that the nestin+ and nestin- cholinergic neurons projected to hippocampus**. (A) Photomicrograph demonstrating the deposition of fast blue dye throughout the entire MS/DBB area and the location of ChAT+ and nestin+ neurons. (B) The ChAT+ neurons in MS/DBB area. (C) The nestin+ neurons in the MS/DBB area. (D) The fast blue transported from the hippocampus was localized in the neurons of the MS/DBB area. (E) The double immunostaining of ChAT+ neurons and nestin+ neurons, arrows point to the double labelling neurons (nestin+ cholinergic neurons). (F) Photomicrograph of neurons labelled by retrograde tracing of fast blue from the hippocampus and nestin+/nestin- cholinergic neurons. Arrows and arrowheads point to the nestin+ and nestin- cholinergic neurons labelled with fast blue. Scale bar: 50 μm in A; 50 μm in B-F.

Examination of serial section of the basal forebrain region 5 days after injection of the fast blue revealed that the blue colour fluorescence could be visualized clearly. The fast blue labelled somas were seen throughout the entire MS/DBB region. Histological examination of the MS/DBB area after labelling revealed striking intense signals in the cell body, however, the neuritis were difficult to distinguish from background. In order to define the anatomical circuits of the nestin+ and nestin- cholinergic projection to the hippocampus, we evaluated the percentage of ChAT and nestin immunoreactivity among the fast blue-labelled neurons in the MS/DBB region after fast blue intra-hippocampus instillation. The nestin and ChAT immunoreactive cells were clearly labelled by green and red colour fluorescence specifically. In order to find the ratio of the nestin+ or nestin- cholinergic neurons projection to the hippocampus, the double or triple fluorescence of combined immunohistochemistry and retrograde labelling were carefully measured. Approximately 20.40% of the fast blue-labelled neurons in the MS/DBB area were ChAT-immunoreactive. In which, 48.04% were nestin-expressing neurons, and 51.96% nestin non-expressing cholinergic neurons (Figure 10).

## Discussion

The main findings of this study were as follows: the electrophysiologically recorded cells expressing nestin mRNA in the MS/DBB are functional neurons; the majority of nestin+ neurons are cholinergic neurons rather than GABAergic or glutamatergic neurons; nestin+ & ChAT+ neurons are more excitable and received stronger excitatory synaptic afferent currents than those of the nestin- & ChAT+ neurons. In addition, the fast blue retrograde labelling experiment demonstrates that both the nestin+ and nestin- cholinergic neurons sent projections to hippocampus.

### The recorded cells expressing nestin in MS/DBB are functional neurons

Using whole-cell recording combined with sc-RT-PCR, our experiment demonstrated that all of the electrophysiologically recorded cells expressing nestin mRNA in MS/DBB were excitable. Whole cell currents of functional neurons could be elicited from these neurons in voltage-clamp mode; typical neural action potential was observed in response to a short period of depolarization, and repetitive action potentials could be elicited when sustained positive current was applied in current-clamp mode. Due to their typical intrinsic membrane properties recorded here, we concluded that these cells are functional neurons rather than stem cells or glial cells, since neural stem cells and glial cells could not be excited to produce neural action potential. For the first time, these data confirmed that nestin+ cells in the MS/DBB are functional neurons by the joint evidence of mRNA expression and electrophysiological properties from whole-cell recordings.

### Most of the nestin+ neurons in MS/DBB were cholinergic neurons

Sc-RT-PCR results revealed 87.5% of nestin+ neurons expressed ChAT mRNA, and about 44.3% of ChAT+ neurons expressed nestin mRNA. However, no nestin+ neurons expressed GAD_67 _mRNA or VGLUT mRNA. These results were further confirmed by the nestin and ChAT immunofluorescent labelling of biocytin filled neurons and fast blue retrograded labelled neurons under the laser confocal microscope, which confirmed that nearly one half of the ChAT-ir neurons were nestin-ir neurons. There were a few nestin+ neurons that did not express ChAT+, GAD_67 _or VGLUT mRNA. This may stem from limit of methodology, or there were a new class of neurons in MS/DBB.

Taken together, these observations provide new evidence that the majority of nestin+ neurons in MS/DBB were cholinergic neurons rather than GABAergic or glutamatergic neurons. In other words, cholinergic neurons in the MS/DBB could be subdivided into nestin-expressing cholinergic neurons (NEChs) and nestin non-expressing cholinergic neurons (NNChs). This result is partially inconsistent with the previous reports, in which about 35% of nestin-ir neurons were ChAT-ir [8]. The possible reason of the difference may stem from the sensitivity of the methodology. Previous studies used double-staining immunohistochemistry, which visualized same neuron with 3'-diaminobenzidine (DAB) and 3, 3', 5', 5-tetramethylbenzidine-sodium tungstate (TMB-ST). The two chromogenic reagents may interfere with each other and lead to false-negative results. In addition, TMB colour faded rapidly. Consequently, in the use of this labelling method some neurons were stained with ambiguous colour and it was, therefore, difficult to accurately assess their phenotype. The other possible reason may be that sc-RT-PCR detects mRNA but immunohistochemistry detects proteins, and that not all mRNA may be translated into proteins.

### NEChs and NNChs had different electrophysiological properties

The *I*_h _current serves as a pacemaker, and is implicated in generating rhythmic bursts in a number of brain structures such as thalamus, hippocampus and cortex [25]. Previous work revealed that cholinergic neuron displayed slow-firing and little or no *I*_h_; GABAergic neuron was fast-firing neuron, had a substantial *I*_h_; and glutamatergic displayed electrophysiological properties similar to cholinergic neurons such as the occurrence of a very small *I*_h _[4]. In our study, we found GABAergic neurons had prominent *I*_h_, whereas cholinergic and glutamatergic neurons had small or no *I*_h_, which was consistent with previous studies.

The *I*_h _and sEPSCs amplitude of NEChs were larger than those of NNChs, which implies that NEChs are more excitable than NNChs and may have different roles in learning and memory. The higher sEPSCs frequency and amplitude of the NEChs suggests that the NEChs received stronger spontaneous excitatory synaptic inputs than those of the NNChs. The mEPSCs amplitude of NEChs were significantly larger than those of NNChs, but no differences were observed between the amplitudes of mEPSCs and sEPSCs on NEChs or NNChs, suggesting that presynaptic spontaneous action potential did not affect the sEPSCs amplitude of both the NEChs and NNChs. These data also suggest that the higher sEPSCs amplitude of NEChs was due to more excitatory receptors or higher sensitivity of the receptors compared to the NNChs. The similar multiplicities of NEChs and NNChs suggested that both kinds of neurons share same level of maturity. Interestingly, the mEPSCs frequency of NEChs, but not NNChs, was remarkably lower than sEPSCs frequency, which led to the large shift in the frequency of spontaneous activity, indicating that a great number of spontaneous events of NEChs recorded in absence of TTX could be attributed to the neurotransmitter released by action potential dependent mechanisms. Therefore, the stronger spontaneous excitatory afferent current could be attributed to the higher synaptic transmission efficacy to the NEChs, and higher excitability of the NEChs compared to the NNChs.

### NEChs and NNChs project parallelly to hippocampus

MS/DBB is one of the most important inputs to the hippocampal neurons [26,27]. The hippocampus receives its cholinergic projections predominantly from MS, and to a lesser extent, from the VDB (Mesulam et al., 1983a). This cholinergic input is of particular importance for learning and memory processes (Hasselmo, 1999; Kesner, 1988).

In our experiment, we have demonstrated that nestin+ neurons are a subtype of basal forebrain cholinergic neurons using single-cell RT-PCR, immunohistochemistry and electrophysiological property analysis. Therefore, basal forebrain cholinergic neurons could be divided into two groups according to whether expressing nestin and their electrophysiological properties. Retrograde labelling combined with ChAT and nestin immunofluorescence suggested that both of the nestin+ and the nestin- cholinergic neurons project to the hippocampus. Therefore, we concluded that there are two parallel septo-hippocampal cholinergic pathways. One pathway originates from MS/DBB nestin-expressing cholinergic neurons (NEChs), and the other pathway originates from nestin non-expressing cholinergic neurons (NNChs). Because the NEChs and NNChs had different intrinsic electrophysiological properties and received distinct excitatory synaptic inputs, they may have different functions in maintaining the electrical activities in the hippocampus, which is worthy of further study.

Nestin is expressed transiently by neural progenitor cells and reactivated glial cells [17] and is involved in cell survival and reparation [18]. Previous studies revealed that Purkinje cells in cerebella of Creutzfeldt-Jakob disease and dorsal root ganglia neurons following nerve injury express nestin, and that nestin expression might represent a stage of protective reaction to prolong the survival of neurons or enhance the differentiation of neurons in order to compensate for lost neurons [18,28]. In addition, intracerebroventricular injection of colchicine can lead to irreversible reduction of basal forebrain cholinergic neurons [29], but only cause transient reduction of basal forebrain nestin-ir neurons [30]. As most of the nestin mRNA+ neurons were cholinergic neurons, it is implied that nestin expression might mark a special stage of cholinergic neurons that were relatively spared from severe degeneration and cell death. It is also possible that nestin expression marks a type of newly differentiated neurons that compensate for lost MS/DBB cholinergic neurons. The mechanism underlying the protective plasticity and viability of NEChs and NNChs are worthy of further study using selectively or non-selectively MS/DBB neurons damaging model [31].

## Conclusions

In conclusion, we studied the electrophysiological properties of the novel nestin+ neurons in the MS/DBB, and demonstrated that most of nestin+ neurons are functional cholinergic neurons. We also provided evidence that the NEChs had higher excitability and received stronger spontaneous excitatory synaptic inputs than those of the NNChs. Then we demonstrated that both of the NEChs and NNChs projected to the hippocampus. The different electrophysiological properties of NEChs and NNChs and common neural circuits to hippocampus suggested that there are two parallel septo-hippocampal cholinergic pathways that may have different functions. Whether nestin expression affects the cholinergic neurons' properties require further study by manipulation of gene expression. These results will not only facilitate our understanding of the structures and biological function of basal forebrain, the mechanism of learning and memory, the ageing process and the pathology of AD, but may also provide a new insight for AD treatment.

## Methods

### Ethics Statement

All experiments were approved by Institutional Animal Care and Use Committee of Sun Yat-sen University. All work was carried out in accordance with the National Institute of Health Guide for the Care and Use of Laboratory Animals (NIH Publications No. 80-23) revised 1996. Every effort was made to minimize the animals used and their suffering.

### Brain slice preparation

Brain slices containing the MS/DBB were prepared from 40 Sprague-Dawley rats (14-21 days postnatal) of either sex as previously described [4]. Briefly, rats were killed by decapitation, and brains were quickly removed and submerged in ice-cold artificial cerebrospinal fluid (ACSF) containing (in mM): 126 NaCl, 3 KCl, 1.25 NaH_2_PO_4_, 2.0 MgSO_4_, 2.0 CaCl_2_, 24 NaHCO_3_, and 10 D-glucose, equilibrated with 95% O_2_-5% CO_2_, pH 7.4. After cooling in the ACSF, brain was trimmed with a razor blade and fixed to specimen tray of a vibratome (Vibratome 3000 EP sectioning system, USA) with cyanoacrylate adhesive. Coronal slices of 400 μm thickness containing the MS/DBB were prepared and transferred to a custom-designed holding chamber allowed to recover in oxygenated ACSF at 32 ± 0.5°C for 30 min, and then put at room temperature (25 ± 1°C ) for additional 1- 4 hour before experimental recordings. After 1-1.5 hour, the slice was transferred to a Plexiglas recording chamber on the stage of a Nikon microscope (Eclipse FN1, Japan) and continuously perfused with oxygenated ACSF at a rate of 1-2 ml/min for electrophysiological recordings. The recording chamber was kept at a temperature of 26 ± 0.5°C by an automatic temperature controller (WARNER TC-324B, USA). Neurons in the slice were visualized using infrared differential interference contrast (IR/DIC) video microscopy (IR-1000E, USA). Medium to large sized neurons in MS/DBB that had full form and clear outline were selected randomly for patch recording.

### Whole-cell recordings

Patch pipettes were made from diethyl pyrocarbonate (DEPC, Amresco, USA) processed and heat-sterilized borosilicate glass capillary tubing containing a filament (OD: 1.5 mm, ID: 0.86 mm) by Sutter P-97 horizontal puller (Sutter Instruments Co., Novato, USA). The pipette filled with 5μl internal solution had 3-6 Mohm pipette resistance. The internal solution was prepared with nuclease-free water containing (in mM): 144 potassium gluconate, 3 MgCl_2_, 10 Hepes, 0.2 EGTA, 2 K_2_-ATP, 0.3 Na_3_-GTP, pH 7.2 (285-295 mOsm). Whole-cell recordings in voltage-clamp and current-clamp modes were performed at 26 ± 0.5°C using an Axon Multi Clamp-700B amplifiers and a Digidata 1322A analog-to-digital converter (Axon Instruments Inc, USA). The output signal was continuously filtered at 3 kHz, digitized at a sampling rate of 20 kHz, and stored in a computer for off-line analysis using pClamp 9.2 software (Axon instruments Inc, USA) or Mini Analysis 6.0.3 (Synaptosoft, Leonia, NJ, USA) software.

### Intrinsic membrane properties analysis

**Figure 11:**
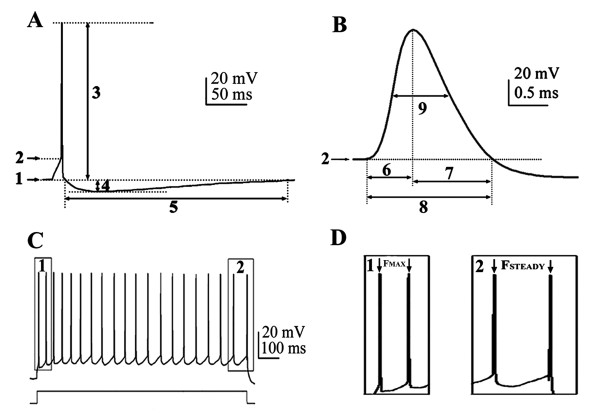
**Measurement of action potential parameters and firing patterns**. A, B: Measurement of action potential parameters. 1) Resting membrane potential (mV); 2) Action potential threshold (mV); 3) Action potential amplitude (mV); 4) After hyperpolarization (AHP) amplitude (mV); 5) AHP duration (ms); 6,)Spike rise time (ms); 7) Spike decay time (ms); 8) Spike duration; 9), Spike half width (ms). C, D: Measurement of parameters of firing properties of a medial septal and diagonal band complex neuron subjected to a depolarizing current pulse: time interval between: 1, the first two action potentials; 2, the last two action potentials. Reciprocal of time intervals gives maximum firing frequency (F_MAX_) and steady firing frequency (F_STEADY_). Scale bars: 20 mV, 50 ms in A; 20 mV, 0.5 ms in B; 20 mV, 100 ms in C.

All electrophysiological recordings were made from the medial septum and the vertical limb of the diagonal band of Broca. The resting membrane potential of each neuron was measured in current-clamp mode (I = 0) just after passing in whole cell configuration. All membrane potentials were corrected for junction potential (about -15 mV). The experiment was continued only when the resting membrane potential was more negative than -45 mV, spikes overshot 0 mV and the series resistance was less than 30 Mohm [4]. In current-clamp mode, the membrane potentials of selected MS/DBB neurons were held at - 60 mV or - 80 mV, and a series of hyperpolarizing and depolarizing current pulses (0.1 - 0.3 nA, 1- 4 s duration) were applied from both holding potentials to determine membrane properties and firing properties. Action potential properties were analyzed from membrane response to a short threshold depolarizing current pulse (10 ms, 0.1 - 0.3 nA) applied at -60 mV [14,32] using standard criteria (Figure 11, A and 11B) [33,34].

Firing patterns were analyzed in response to series of 1-4 s depolarizing current pulses applied from - 60 mV, and repeated from a membrane potential of -80 mV. Firing frequencies were determined for each neuron depolarized from -60 mV using a current pulse of 100-300 pA. Maximal firing frequency (F_MAX_) and steady firing frequency (F_STEADY_) were calculated from the time interval between the first two spikes or the last two spikes respectively in a train of action potentials evoked by a 1 s depolarizing current pulse, and mean firing rate was calculated from the number of spikes evoked by a 1 s depolarizing current pulse (Figure 11, C and 11D). Spike adaptation was measured using [(F_MAX _- F_STEADY_)/F_MAX_].

The voltage-dependent inward rectification (depolarizing sag) was determined by application of a series of 4 s hyperpolarizing current from -60 mV, and its amplitude was measured for the current pulse inducing an initial hyperpolarization to - 95 mV. The hyperpolarization activated current (*I *_h_) was activated using a series of 2 s long hyperpolarizing voltage steps from -50 to -120 mV in 10 mV increments. The currents evoked by injection of hyperpolarizing voltage steps presented two components: instantaneous current (an initial instantaneous change in membrane conductance) and steady-state current (a secondary slowly developing inward current). The amplitude of *I*_h _was measured by subtracting instantaneous current from steady-state current, and was represented by plotting the instantaneous and steady state currents as a function of membrane voltage.

### Analysis of the excitatory synaptic input current properties

The selected neurons were clamped at -70 mV. The internal solution was the same as that used in the experiment for intrinsic membrane properties study. To study spontaneous excitatory postsynaptic currents (sEPSCs), bicuculline (10 μM) was added to the perfusing solution to block GABA_A_-mediated synaptic transmission. Tetrodotoxin (TTX, 1 μM) was added to the bicuculline-containing solution to obtain mEPSCs. The output signal was continuously filtered at 3 kHz, digitized at a sampling rate of 20 kHz, and stored in a computer for off-line analysis using Mini Analysis 6.0.3 (Synaptosoft, Leonia, NJ, USA) software. The excitatory postsynaptic currents analysis was based on 180 s gap free recordings. The detection threshold was set at twice the baseline noise. The fact that no false events would be identified was confirmed by visual inspection for each synaptic current. The cells were rejected if the access resistance changed more than 20% during the experiment. The addition of 10 μM 6-cyano-7-nitroquinoxaline-2, 3-dione (CNQX, a non-NMDA glutamate receptor antagonist) abolished all synaptic events, indicating the involvement of non-NMDA glutamate receptors (data not shown).

To compare the synaptic connectivity, we calculated multiplicity index, a common parameter used as a maturation index in synaptic developmental studies, defined as the number of release sites per presynaptic neuron could release neurotransmitter simultaneously onto the recorded cell [35-37]. Multiplicity index was calculated as the mean amplitude of action-potential-driven events divided by mean quantal size (q: mean amplitude of mEPSCs). The Multiplicity index was calculated as follows:

$$\text{Multiplicity}=({\text{V}}_{\text{b}}.\text{b}-{\text{V}}_{\text{q}}.\text{q})/[({\text{V}}_{\text{b}}-{\text{V}}_{\text{q}}).\text{q}]$$

Where V_b _and V_q _are the mean frequency of sEPSCs and mEPSCs; b and q are the mean amplitude of sEPSCs and mEPSCs.

### Cytoplasm harvest and reverse transcription

Cytoplasm harvest and reverse transcription (RT) were performed as previously described [4,38-40] with a little change according to the instruction of the Primescript^TM ^RT-PCR kit (Takara Biotechnology, Dalian, China). Briefly, after electrophysiological recording, the cell Cytoplasm was aspirated by a gentle negative pressure in the patch pipette under visual control. The series resistance and leak currents were monitored throughout the aspiration procedure and the negative pressure was stopped before the seal was lost. The pipette was then quickly removed from the slice, and its content was expelled into a 0.2 ml PCR tube containing 5 μl of 20 mM dithiothreitol (DTT, Amresco, USA), 20 U ribonuclease inhibitor and 20 picomoles of random hexamers (volume was measured and adjusted to 10 μl with nuclease-free water and quickly cooled on ice). For first strand cDNA synthesis, the tube was heated to 95°C for 1 min and quickly cooled on ice. 100 U Primescript^TM ^RTase, 4 μl 5 × First Strand Buffer, 1 μl dNTPs (10 mM each), 1 μl 0.1 M DTT, 20 U ribonuclease inhibitor and 3 μl RNase free dH_2_O were then added to make the total reaction volume to 20 μl. The RT was initiated at 30°C for 10 min, continued for 2 h at 42°C and overnight at 37°C. The reverse transcriptase was denaturized at 70°C for 15 min and RNA was removed by incubation with 2 U ribonuclease H (1 μl, Takara Biotechnology, Dalian, China) for 20 min at 37°C.

### Multiplex PCR

The cDNAs for ChAT, glutamic acid decarboxylases _67 _(GAD_67_), vesicular glutamate transporters 1 and 2 (VGLUT_1 _and VGLUT_2_), and nestin were amplified with a two-step PCR protocol, slightly modified from that described by Sotty et al. (2003) and Puma et al. (2001). Target cDNAs were amplified simultaneously as a multiplex PCR in the first round and amplified individually in the second round PCR with nested primers. The first round PCR reaction was performed in a Mastercycler (Eppendorf, Germany) with a final volume of 100 μl containing the 21 μl RT reaction, 1× PCR Buffer (50 mM KCl, 10 mM Tris-HCl, 1.5 mM MgCl2, pH 8.3), 50 μM of each of the dNTPs, 10 p mol of each selected primer and 2.5 U TaKaRa Ex Taq^TM ^HS polymerase (Takara Biotechnology, Dalian, China). The PCR amplification protocol was as follows: 2 min at 94°C; 20 cycles: 30 s at 94°C, 30 s at 60°C, 35 s at 72°C; 5 min at 72°C. Second-round PCRs were performed individually in reaction volume of 25 μl, using 2 μl of the first round PCR product and specific primer pair by 35 PCR cycles under similar conditions as first round PCR but the concentration of dNTPs was 200 mM. Five microliters of each last-round PCR product was run on a 1.5% TEA agarose gel stained with ethidium bromide, using a 100 bp DNA ladder as molecular weight marker. Some PCR products had been sent to professional company (Beijing AuGCT biotechnology Co., ltd, Beijing, China) for automatic sequencing to determine their specificity. Primers used in the experiment are shown in Table 2. To ensure the primer specificity all primers were designed to span multiple intron/extron boundaries. To rule out the false-positive response, bathing medium near the recorded neuron was collected using a standard recording pipette to substitute the cytoplasm as a media control, and double distilled water substituted the cytoplasm was used as blank control, the same RT-PCR reactions were performed. Experiment controls for single cell RT-PR are made at each harvesting session, only the data from experiments in which both media control and blank control were negative could be taken into account, otherwise the cell would be discarded [39,41].

**Table 2:** Primer sequences used in the experiment

mRNA	Accession no.^a^	primer sequence	Start position ^b^	Product size^c^
ChAT (first round)	XM_001061520	CAGGAAGGTCGGGTGGACAACATC TCCTTGGGTGCTGGTGGCTTG	1675 2198	524 bp
GAD_67_ (first round)	NM_017007	TTTGGATATCATTGGTTTAGCTGGCGAAT TTTTTGCCTCTAAATCAGCTGGAATTATCT	762 1162	401 bp
VGLUT_1_ (first round)	NM_053859	TACTGGAGAAGCGGCAGGAAGG CCAGAAAAAGGAGCCATGTATGAGG	188 498	311 bp
VGLUT_2_ (first round)	NM_053427	CCCGCAAAGCATCCAACCA TGAGAGTAGCCAACAACCAGAAGCA	1264 1682	419 bp
Nestin (first round)	M34384	CTCGGGAGTGTCGCTTAGAG ATTAGGCAAGGGGGAAGGGA	783 1216	434 bp
ChAT (second round)	XM_001061520	ATGGCCATTGACAACCATCTTCTG CCTTGAACTGCAGAGGTCTCTCAT	1840 2163	324 bp
GAD_67_ (second round)	NM_017007	CTGACATCAACTGCCAATACCAA GGAGAAAATATCCCATCACCATC	793 929	137 bp
VGLUT_1_ (second round)	NM_053859	GTGGTGGACTGCACTTGCTT CATGTATGAGGCCGACAGTCTC	274 484	211 bp
VGLUT_2_ (second round)	NM_053427	CGCAAAGCATCCAACCAT TGTGGGACCGCAGACAAC	1266 1529	264 bp
Nestin (second round)	M34384	GGA GCA GGA GAA GCA AGG GGT CCA GAA AGC CAA GAG	931 1115	185 bp

ChAT, choline acetyltransferase; GAD, glutamic acid decarboxylases; VGLUT, vesicular glutamate transporter. ^a ^The GenBank accession number for the cDNA sequence used in primer design; ^b ^The cDNA position of the 5'' end of primer; ^c ^Expected PCR product length.

### Immunohistochemistry and laser confocal scanning of recorded neurons

For subsequent immunofluorescence study of the recorded cells, biocytin (0.2%) was added to internal solution. In the cells from which recordings had been made, biocytin, nestin and ChAT were visualized by triple fluorescence: Rhodamine Red was the fluorescent label for biocytin, cy2 for nestin-ir and Alexa 405 for ChAT-ir. After whole-cell recording, the brain slices were fixed by immersion in 4% paraformaldehyde in 0.1 M phosphate buffer, pH 7.2, at 4°C for l-2 h, washed in 0.1 M phosphate buffer, and cryoprotected by immersion in 30% buffered sucrose at 4°C for several hours. The following day, serial sections at 50 μm thickness were cut on a cryostat, and collected in 0.01 M phosphate-buffered saline (PBS). Sections were washed (3 × 10 min) in 0.01 M PBS followed by incubation in 1% bovine serum albumin (containing 0.3% Triton X-100 in PBS) for 30 min to prevent non-specific conjugate binding. Sections were incubated with monoclonal mouse anti-nestin antibody (1:800, Rat-401, Pharmingen) and polyclonal rabbit anti-ChAT antibody (1:1000, Chemicon) for 2 h at 37°C and then for 14 h at 4°C. After a 3 × 10 min rinse in PBS, the sections were incubated in a cocktail containing Rhodamine Red X-conjugated streptavidin (1:5000, Jackson Immuno Research Laboratories), a cy2-conjugated goat anti-mouse antibody (1:200, Jackson Immuno Research Laboratories) and a Alexa 405-conjugated goat anti-rabbit antibody (2 μg/ml, Invitrogen) for 2 h at room temperature [42].

In control experiments, the primary antibodies were omitted and, as expected, this resulted in the absence of any cellular labelling. Additional controls were performed by switching the fluorochromated immunoreagents related to the markers visualized by triple fluorescence labelling procedures which resulted in identical staining patterns.

All sections were then analyzed with the LSM 510 Meta (Zeiss). Nestin-ir was observed with the blue FITC filter; ChAT-ir was viewed with the DAPI filter; biocytin-filled cell was viewed with the Rhodamine Red filter. Confocal images were scanned, edited, reconstructed and measured using LSM 510 Meta software (Zeiss).

### Analyses of the projections of nestin+ and nestin- cholinergic neurons

To demonstrate whether the nestin+ and nestin- cholinergic neurons projected to hippocampus, retrograde labelling combined with double immunostaining protocols were used. Four rats were instilled with 0.5 μl of 3% fast blue dye into the CA1 area (AP-3.8, L 1.5, H 3.0) of the hippocampus. After a 5-d transport period, the experimental animals were sacrificed, immunofluorescence of nestin and ChAT were performed with a standard protocols. Rhodamine Red was the fluorescent label for ChAT-ir, cy2 for nestin-ir neurons [42]. The localization of fast blue in nerve cell bodies of the MS/DBB area were observed, counted, and analyzed. The experimental controls were performed as described above.

### Statistical analysis

All statistical analyses were performed with SPSS 11.5 for Windows. Data were analyzed statistically using either the student's *t*-test, one-way ANOVA (post hoc multiple comparison by least-significant difference (LSD) or Dunnett's T3 test), or the Kolmogorov-Smirnov test. Significance level for all measures was set at *P *< 0.05. Data are presented as means ± S.E.M (standard error of the mean). All figures were prepared using CorelDraw 10.0 (Corel; Ottawa) and Adobe Photoshop CS (Adobe; San Jose, CA).

## Competing interests

The authors declare that they have no competing interests.

## Authors' contributions

JHZ designed and carried out the study, performed the statistical analysis, and drafted the manuscript. HYG participated in the design of the study, performed the statistical analysis, and helped to draft the manuscript. ZBY conceived the study, and participated in its design and coordination, and helped to draft the manuscript. JTZ carried out the molecular genetic studies, participated in the sequence alignment. KHG participated in the immunohistochemistry and neuronal projection study. DPL participated in the design of the study and performed the statistical analysis. TMG participated in design and coordination of the study, and helped to draft the manuscript. All authors read and approved the final manuscript.
